# An Enzyme-Free Sandwich Amperometry-Type Immunosensor Based on Au/Pt Nanoparticle-Functionalized Graphene for the Rapid Detection of Avian Influenza Virus H9 Subtype

**DOI:** 10.1186/s11671-022-03747-8

**Published:** 2022-11-21

**Authors:** Jiaoling Huang, Zhixun Xie, Meng Li, Sisi Luo, Xianwen Deng, Liji Xie, Qing Fan, Tingting Zeng, Yanfang Zhang, Minxiu Zhang, Sheng Wang, Zhiqin Xie, Dan Li

**Affiliations:** Guangxi Key Laboratory of Veterinary Biotechnology, Key Laboratory of China (Guangxi)-ASEAN Cross-Border Animal Disease Prevention and Control, Ministry of Agriculture and Rural Affairs of China, Guangxi Veterinary Research Institute, 51 You Ai North Road, Nanning, 530001 Guangxi China

**Keywords:** Electrochemical immunosensor, Au/Pt nanoparticles, Electrocatalysis, Avian influenza virus H9 subtype

## Abstract

Avian influenza virus H9 subtype (AIV H9) has contributed to enormous economic losses. Effective diagnosis is key to controlling the spread of AIV H9. In this study, a nonenzymatic highly electrocatalytic material was prepared using chitosan (Chi)-modified graphene sheet (GS)-functionalized Au/Pt nanoparticles (GS-Chi-Au/Pt), followed by the construction of a novel enzyme-free sandwich electrochemical immunosensor for the detection of AIV H9 using GS-Chi-Au/Pt and graphene–chitosan (GS-Chi) nanocomposites as a nonenzymatic highly electrocatalytic material and a substrate material to immobilize capture antibodies (avian influenza virus H9-monoclonal antibody, AIV H9/MAb), respectively. GS, which has a large specific surface area and many accessible active sites, permitted multiple Au/Pt nanoparticles to be attached to its surface, resulting in substantially improved conductivity and catalytic ability. Au/Pt nanoparticles can provide modified active sites for avian influenza virus H9-polyclonal antibody (AIV H9/PAb) immobilization as signal labels. Upon establishing the electrocatalytic activity of Au/Pt nanoparticles on graphene towards hydrogen peroxide (H_2_O_2_) reduction for signal amplification and optimizing the experimental parameters, we developed an AIV H9 electrochemical immunosensor, which showed a wide linear range from 10^1.37^ EID_50_ mL^−1^ to 10^6.37^ EID_50_ mL^−1^ and a detection limit of 10^0.82^ EID_50_ mL^−1^. This sandwich electrochemical immunosensor also exhibited high selectivity, reproducibility and stability.

**Supplementary Information:**

The online version contains supplementary material available at 10.1186/s11671-022-03747-8.

## Introduction

Since avian influenza virus H9 subtype (AIV H9) was isolated from affected chickens in 1994, AIV H9 has become widespread in poultry [1, 2]. AIV H9 mainly causes poor weight gain, difficulty breathing and reduced egg production, leading to substantial economic losses in the poultry industry [3]. Therefore, rapid, sensitive and specific assays are urgently needed to screen for and control AIV H9 infection.

In recent years, electrochemical immunosensors have attracted increasing attention in clinical diagnosis due to their simple operation, rapid response, low instrumentation cost, excellent sensitivity of electrochemical techniques and high specificity of immunoreactions. In particular, sandwich-type electrochemical immunosensors have been widely applied in clinical diagnosis because double signal amplification with the substrate material and detection antibody labels enhance the electrochemical immunosensor sensitivity [4, 5]. The signal amplification strategy is the most important factor for developing sandwich-type electrochemical immunosensors, and it largely relies on employing various signal amplification labels in combination with secondary antibodies to form conjugated immunocomplexes [6–8]. Nanomaterials have attracted considerable research interest because they not only show enzyme-mimetic activity but also have some improved properties over native enzymes, such as good stability, easy synthesis and cost effectiveness [9, 10]. Among the variety of nanomaterials, noble metal nanostructures have been widely used as labels to fabricate enzyme-free electrochemical immunosensors due to their excellent stability, high electrocatalytic activity, simple synthesis and easy storage [11–13]. In particular, Au and Pt nanoparticles have aroused wide interest due to their excellent electrochemical catalytic properties towards the reduction of H_2_O_2_, and thus, Au and Pt nanoparticles have been used to electrochemically catalyse the reduction of H_2_O_2_ and increase the sensitivity of electrochemical immunosensors [14–16]. Moreover, bimetallic nanomaterials usually show much higher catalytic activity than their monometallic counterparts because of the synergistic effect [17, 18].

A graphene sheet (GS) is a material with a two-dimensional monolayer carbonaceous structure that shows remarkable mechanical stiffness, a high specific surface area, high thermal conductivity and fast electron transport and has been utilized in different applications in various fields, including biomedical applications, energy conversion and storage systems, electrocatalysts and disease diagnosis [19–23]. GSs are usually used as electrode modification materials and signal amplifier materials in electrochemical sensor applications because of their unique physicochemical and biological properties [24–26]. Nonetheless, GSs displayed poor hydrophilicity and difficulty immobilizing biometric molecules, which generally adversely affect the performance of electrochemical sensors. Hence, GSs have been modified with organic molecules or inorganic molecules to avoid this problem. We have found that GS-chitosan (GS-Chi) nanocomposites are easily immobilizing biometric molecules and are better dispersed in water [27]. Interestingly, Chi is rich in amino groups [28], and GS-Chi might improve the loading capacity and dispersion of Au/Pt through covalent binding between noble metal nanoparticles and amino (-NH_2_) groups. In addition, Au/Pt might also enable the facile conjugation of capture antibodies due to the formation of stable Au–N and Pt–N bonds between Au/Pt and –NH_2_ residues on antibodies [29]. Therefore, the combination of Au/Pt and GS-Chi may ideally enhance the signal response of the electrochemical immunosensor.

Substrate materials with effective immobilization of capture antibodies and high conductivity to facilitate transfer of electrons between the electrode and electrolyte are another important factor to improve the quality of electrochemical immunosensors. The GS-Chi nanocomposite was employed as the electrode substrate material. Due to the large specific surface area of the GS-Chi nanocomposite, more capture antibodies were stably immobilized onto the surface of the GS-Chi nanocomposite via glutaraldehyde. The superior conductivity of the GS-Chi nanocomposite enhanced electron transfer from the electrolyte to the electrode surface, further enhancing the signal response of the electrochemical immunosensor [28].

In the present study, a GS-Chi nanocomposite was used as a substrate material to immobilize capture antibodies (AIV H9/MAbs) and as a carrier to load catalytically active material (Au/Pt) and detection antibodies (AIV H9/PAbs) to fabricate an enzyme-free sandwich-type electrochemical immunosensor for AIV H9 detection. Due to its very large specific surface area and excellent electrical conductivity, GS-Chi not only increased the amounts of AIV H9/MAbs, Au/Pt and AIV H9/PAbs immobilized but also improved the electron transfer efficiency. A substantial increase in sensitivity towards the reduction of H_2_O_2_ was obtained with the synergetic effect of GS-Chi-Au/Pt. The established electrochemical immunosensor exhibited excellent analytical performance for AIV H9 detection and could potentially be applied as an electrochemical immunosensor for the detection of other pathogens.

## Materials and Methods

### Reagents and Materials

Chloroplatinic acid hexahydrate (H_2_PtCl_6_·6H_2_O), chloroauric acid tetrahydrate (HAuCl_4_·4H_2_O) and bovine serum albumin (BSA) were obtained from Sigma‒Aldrich Chemical Co. (St. Louis, MO, USA). Graphite powder (< 45 mm), NaNO_3_, H_2_SO_4_ and KMnO_4_ were supplied by Guoyao Group Chemical Reagents Co., Ltd. (Shanghai, China). All chemicals were analytical reagent grade. Double-distilled deionized water (ddH_2_O) prepared with a Millipore water purification system was used throughout the experiments. Phosphate-buffered saline (PBS, pH 7.0), which was prepared with 10 mmol/L NaH_2_PO_4_ and 10 mmol/L Na_2_HPO_4_ and containing 0.9% NaCl, was used as washing buffer. Electrolytes with different pH values were prepared by mixing different volumes of 10 mmol/L NaH_2_PO_4_ and 10 mmol/L Na_2_HPO_4_ and contained 0.1 mol/L KCl. Viral transport medium was prepared with PBS containing 5% (v/v) foetal bovine serum, streptomycin (10 mg/mL), kanamycin (10 mg/mL), gentamycin (10 mg/mL) and penicillin (10,000 units/mL).

### Viruses and Antibodies

The AIVs used in this study included AIV subtypes H9, H1, H2, H3, H4, H5 (H5N9 subtype), H6, H7, H8, H10, H11, H12, H13, H14, H15 and H16. All the viruses were collected and stored in a − 80 °C freezer in our laboratory prior to use (Table 1). AIV H9/PAbs and AIV H9/MAbs were prepared by our group [30].

**Table 1:** Viruses used in this study

Avian pathogen	Source	Viral titre
A/Duck/Guangxi/030D/2009 (AIV H1)	GVRI	10^5.61^ EID_50_ mL^−1^
A/Duck/HK/77/76 d77/3 (AIV H2)	UHK	10^6.29^ EID_50_ mL^−1^
A/Chicken/Guangxi/015C10/2009 (AIV H3)	GVRI	10^7.43^ EID_50 _mL^−1^
A/Duck/Guangxi/070D/2010 (AIV H4)	GVRI	10^5.45^ EID_50_ mL^−1^
Inactivated A/Chicken/QT35/98 (AIV H5)	PU	128 HAUs
A/chicken/Guangxi/121/2013 (AIV H6)	GVRI	10^6.19^ EID_50_ mL^−1^
A/chicken PA/3979/97 (AIV H7)	PU	256 HAUs
A/Turkey/Ontario/6118/68 (AIV H8)	UHK	10^6.23^ EID_50_ mL^−1^
A/chicken/Guangxi/116C4/2012 (AIV H9)	GVRI	10^6.37^ EID_50_ mL^−1^
A/Duck/HK/876/80 (AIV H10)	UHK	10^5.73^ EID_50_ mL^−1^
A/Duck/PA/2099/12 (AIV H11)	PU	10^6.47^ EID_50_ mL^−1^
A/Duck/HK/862/80 (AIV H12)	UHK	10^7.12^ EID_50_ mL^−1^
A/Gull/Md/704/77 (AIV H13)	UHK	10^5.37^ EID_50_ mL^−1^
A/Mallard/Astrakhan/263/82 (AIV H14)	UCONN	10^6.72^ EID_50_ mL^−1^
A/Shearwater/Western Australia/2576/79 (AIV H15)	UCONN	10^5.81^ EID_50_ mL^−1^
A/Shorebird/Delaware/168/06 (H16N3)	CIVDC	10^5.63^ EID_50_ mL^−1^

*GVRI* Guangxi Veterinary Research Institute, *UHK* University of Hong Kong, China, *PU* Pennsylvania State University, USA, *UCONN* University of Connecticut, USA, *CIVDC* China Institute of Veterinary Drug Control, *HAUs* Haemagglutination units

### Apparatus

Fourier-transform infrared (FT-IR) spectra were measured with a Nicolet SI10 FT-IR Spectrometric Analyser (USA) using KBr pellets. All electrochemical measurements were performed using a PARSTAT 4000A instrument (Princeton Applied Research, USA). A standard three-electrode system composed of a modified glassy carbon electrode (GCE, Ø = 3 mm) as the working electrode, a saturated calomel electrode (SCE) as the reference electrode and a platinum wire electrode as the counter electrode was used. The nanomaterials were characterized using transmission electron microscopy (TEM) and energy-dispersive X-ray spectroscopy (EDS) elemental analysis (Tecnai G2 F30 S-TWIN, FIE, USA).

### Synthesis of GS-Chi-Au/Pt-AIV H9/PAbs

First, GSs were prepared using the method reported by Hummers, with some modifications [31]. Briefly, 1.0 g of graphite powder and 2.5 g of NaNO_3_ were added to 100 mL of concentrated H_2_SO_4_ and stirred for 2 h at room temperature. Then, 5 g of KMnO_4_ were slowly added to this mixture with continuous stirring and cooled with ice at the same time. The mixture was reacted at 35 °C for 24 h with continuous stirring. Then, 100 mL of ddH_2_O was slowly added to the mixture and stirred at 80 °C for 3 h, and then, 300 mL of ddH_2_O was added. Subsequently, 6 mL of 30% H_2_O_2_ was added to the mixture, many bubbles appeared, and the mixed solution immediately turned bright yellow. The resulting solution was stirred for 3 h, and then, the supernatant was decanted after precipitation for 24 h at room temperature. The obtained yellow slurry was washed with 500 mL of 0.5 mol/L HCl and centrifuged. Next, the slurry was washed with ddH_2_O by centrifugation until the pH of the supernatant was approximately 7.0. Then, 100 mL of ddH_2_O was added and ultrasonicated for 2 h, and GS oxide was obtained. Ten millilitres of 1.0% NaBH_4_ was added dropwise at 95 °C while stirring and incubated for 3 h, and GS oxide was reduced. After centrifugation, the supernatant was decanted, and the precipitate was washed with ddH_2_O three times and then dried in a vacuum drying oven at 90 °C for 8 h to obtain GS.

Second, GS-Chi was prepared using our previously described method [32]. Briefly, 0.05 mg of Chi powder was added to 100 mL of a 1.0% (v/v) acetic acid solution while stirring until it was completely dispersed, and a 0.5 wt% Chi solution was obtained. Then, 100 mg of GSs was added to the 100 mL 0.5 wt% Chi solution, ultrasonicated for 1 h, continuously stirred for 24 h at room temperature, and GS-Chi was obtained.

Third, GS-Chi-Au/Pt was prepared using the following procedure: 1 mL of HAuCl_4_ (10 mmol/L) and 1 mL of K_2_PtCl_4_ (10 mmol/L) were added to 20 mL of the GS-Chi (1 mg/mL) solution, and the mixture was stirred at 25 °C for 3 h. Then, the mixture was heated to 80 °C in a water bath and stirred for 1 h. Au^3+^ and Pt^2+^ were reduced to Au/Pt by Chi at 80 °C. Finally, the GS-Chi-Au/Pt nanocomposite was generated. Different proportions of GS-Chi-Au/Pt were prepared using the method mentioned above by adding different volumes of HAuCl_4_ (10 mmol/L) and K_2_PtCl_4_ (10 mmol/L) to 20 mL of the GS-Chi (1 mg/mL) solution.

**Fig. 1:**
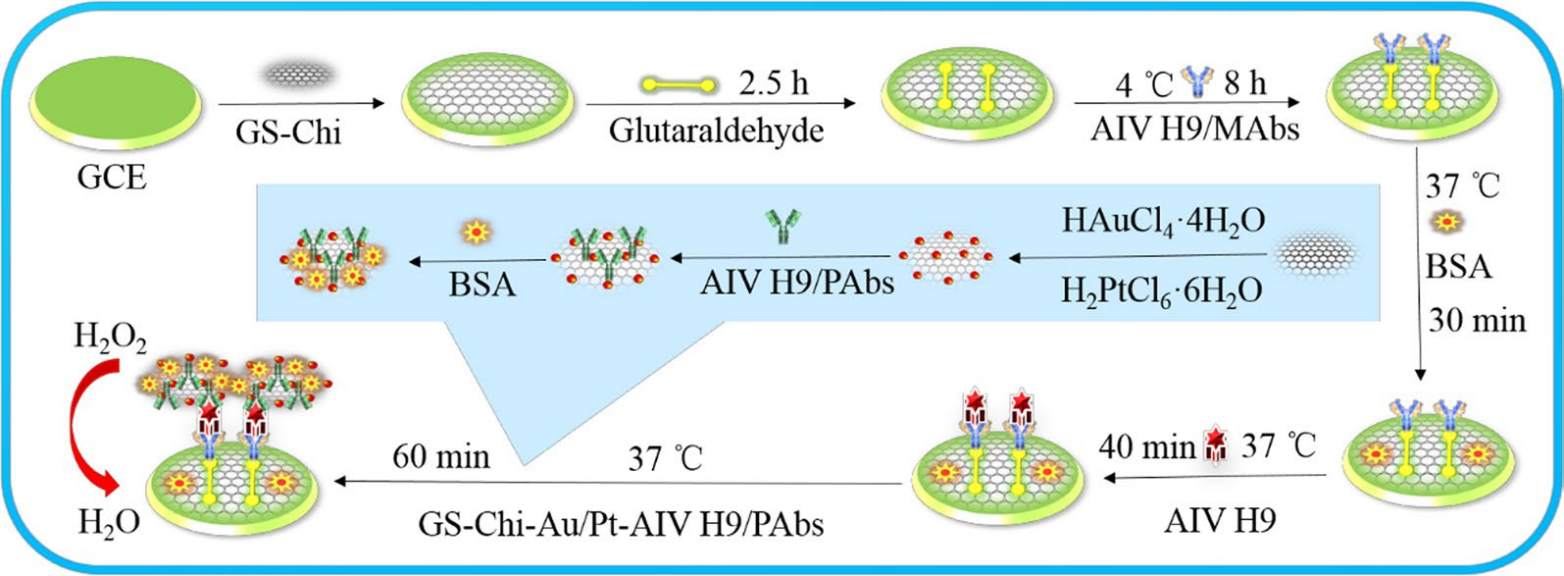
Schematic illustrating the electrochemical immunosensor

Finally, the as-prepared GS-Chi-Au/Pt (10 mL) was mixed with 500 μL of 10 μg/mL AIV H9/PAb (0.5 μg/mL), and the mixture was oscillated in a shaking water bath at 4 °C overnight. The product was centrifuged and washed three times with PBS (pH 7.0) to remove the unbound AIV H9/PAb. The immunocomplex was redispersed into 10 mL of PBS (pH 7.0) containing 1 wt% BSA. Figure 1 shows the procedure used to prepare GS-Chi-Au/Pt-AIV H9/PAbs.

### Fabrication of the Electrochemical Immunosensor

The process used to fabricate the electrochemical immunosensor is shown in Fig. 1. The GCE was successively polished with 1.0 μm, 0.3 μm, and 0.05 μm alumina polishing powders and rinsed with ddH_2_O after each polishing step. The GCE was sonicated in ddH_2_O, ethanol, and ddH_2_O for 5 min each.

Next, 8 μL of GS-Chi (1 mg/mL) was dropped onto the surface of the clean GCE and dried at room temperature. Then, the modified GCE was incubated with 8 μL of 5% glutaraldehyde for 2.5 h and washed with ddH_2_O. Eight microlitres of AIV H9/MAbs (10 μg/mL) was deposited onto the modified GCE and incubated at 4 °C for 8 h. Excess AIV H9/MAbs were removed by washing three times with PBS (pH 7.0). Subsequently, 8 μL of BSA (1 wt%) was dropped on the integrated AIV H9/MAbs-GS-Chi-GCE and reacted for 30 min at 37 °C to block the nonspecific adsorption sites of AIV H9/MAbs-GS-Chi-GCE. Then, the electrode was washed with PBS (pH 7.0) to remove unbound BSA. Finally, the immunosensor was steeped in 1 mL of PBS (pH 7.0) containing 1 wt% BSA and stored at 4 °C when not in use.

### Electrochemical Measurement

First, the immunosensor was incubated with 8 μL of varying concentrations of AIV H9 or clinical samples for 40 min at 37 °C. Second, the unbound AIV H9 and interfering substances were removed by washing the sensor three times with PBS, and 8 μL of GS-Chi-Au/Pt-H9/PAbs was incubated on the immunosensor at 37 °C for 60 min. Finally, the immunosensor was rinsed to remove unbound GS-Chi-Au/Pt-H9/PAbs, and amperometric i–t measurements were performed. We minimized the response of common interfering components and reduced the background current by selecting − 0.4 V as the working potential for the amperometric i–t measurements (Additional file 1: Fig. S1). After the background current was stabilized (approximately 50 s later), 10 mmol/L H_2_O_2_ was added to the electrolyte, unless indicated otherwise, and the current change was monitored and recorded. Electrochemical impedance spectroscopy (EIS) was performed at frequencies ranging from 0.01 Hz to 100 kHz using an amplitude of 10 mV at 0.2 V.

### Clinical Sample Preparation

Oral and cloacal swab samples were gently collected from chickens at different live poultry markets in Guangxi Province and used as clinical samples. The oral swab and the cloacal swab from the same chickens were placed together into a collection tube that contained 1 mL of viral transport medium and counted as a single clinical sample, and the clinical samples were placed in an ice box for transport to our laboratory. Then, cotton swabs were repeatedly shaken and wiped in the transport medium, leading to viral transfer to the transport medium. The cotton swabs were discarded, and the obtained solutions were stored at − 70 °C. Before detection, the samples were repeatedly frozen and thawed 3 times and then centrifuged at 4000 rpm for 5 min. The obtained supernatant was analysed using the fabricated electrochemical immunosensor according to the steps described in “Electrochemical Measurement” section.

## Results and Discussion

### Characterization of the GS-Chi-Au/Pt Nanocomposite

**Fig. 2:**
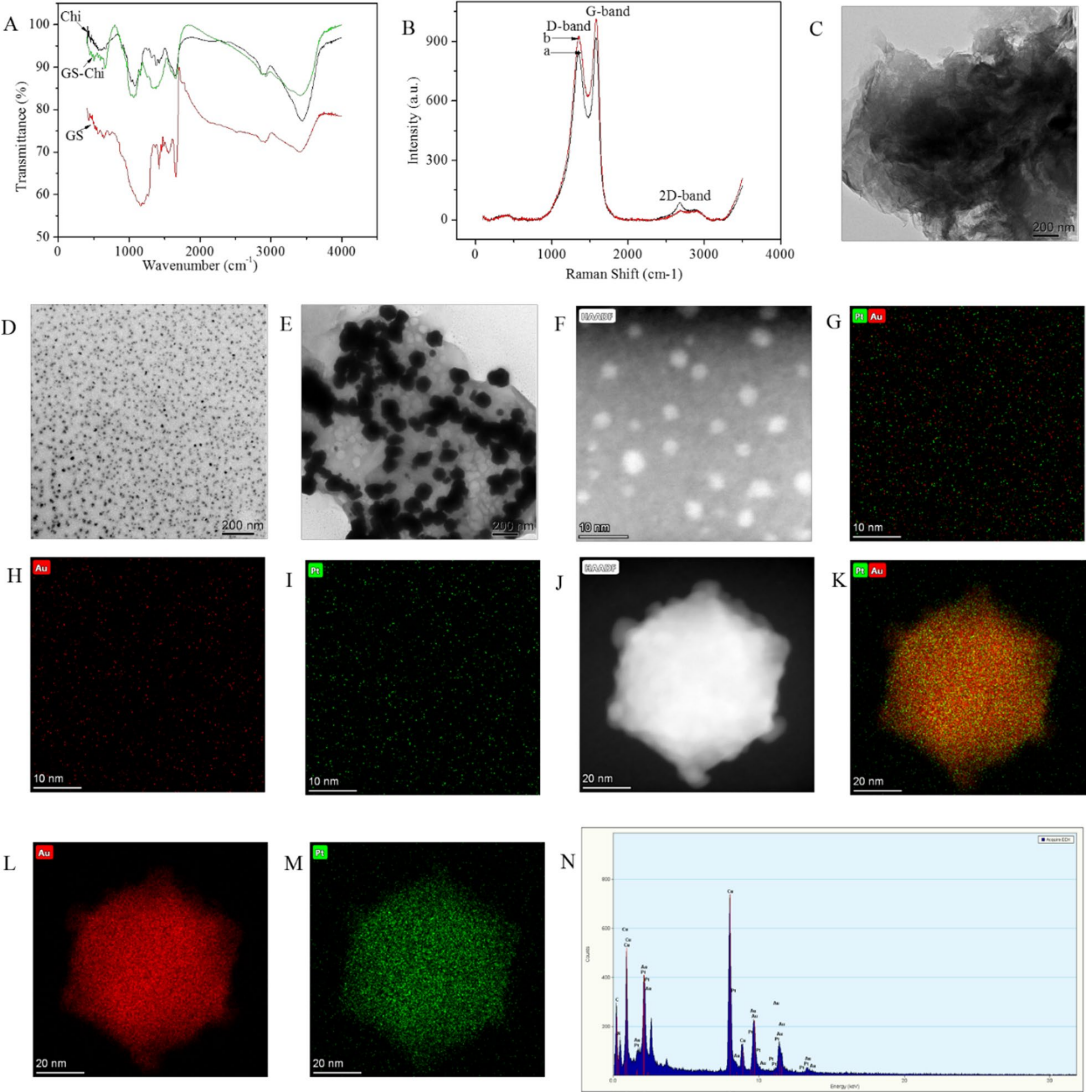
Characterization of the related nanocomposites. **A** FT-IR spectra of Chi, GS, and GS-Chi. **B** Raman spectra of GS (a) and GS-Chi (b). TEM images of GS-Chi (**C**), Chi-Au/Pt (**D**), and GS-Chi-Au/Pt (**E**). HRTEM image of Chi-Au/Pt (**F**). Overlay on the image of Chi-Au/Pt (**G**). Au elemental mapping image of Chi-Au/Pt (**H**). Pt elemental mapping image of Chi-Au/Pt (**I**). HRTEM image of GS-Chi-Au/Pt (**J**). Overlay on an image of GS-Chi-Au/Pt (**K**). Au elemental mapping image of GS-Chi-Au/Pt (**L**). Pt elemental mapping image of GS-Chi-Au/Pt (**M**). EDS elemental analysis of GS-Chi-Au/Pt (**N**)

FT-IR spectroscopy was performed to confirm that GS was modified with Chi. The FT-IR spectrum of Chi presented all of the characteristic bands for Chi, including C–C–O bond stretching vibrations at 1034 cm^−1^, 1081 cm^−1^ and 1154 cm^−1^; –NH_2_ bending vibrations at 1596 cm^−1^ and 1653 cm^−1^; and C–H stretching at 2920 cm^−1^ and 2878 cm^−1^. In addition, –NH_2_ and –OH bond stretching vibrations were observed at 3425 cm^−1^ (Fig. 2A, black line) [33]. Additionally, the FT-IR spectrum of GS preserved all the characteristic bands of GS: benzene ring backbone stretching vibrations at 1420 cm^−1^, 1459 cm^−1^ and 1555 cm^−1^; C=O stretching vibration at 1659 cm^−1^; C–H stretching vibration at 2916 cm^−1^; and O–H stretching vibration at 3406 cm^−1^ (Fig. 2A, green line) [34]. FT-IR spectra of the GS-Chi combination presented all the characteristic bands of GS and Chi, except that the characteristic absorption bands of GS-Chi had different intensities from those of GS and Chi, which indicates that the surfaces of GS were successfully modified with Chi.

Moreover, Raman spectroscopy was employed to confirm the successful synthesis of GS-Chi. As shown in Fig. 2B, the Raman spectrum obtained from GS showed two prominent bands: one was the D-band at 1349 cm^−1^ due to stretching of *sp*^3^ carbons, and the other was the G-band at 1582 cm^−1^ due to stretching of *sp*^2^ carbons. These two peaks were also observed in GS-Chi [35], revealing that GS-Chi still retains the basic structure of GS. In addition, previous studies indicate that the D/G-band ratio changes in the presence of oxygenated groups at the upper and lower surfaces, as well as the edges of sheets. Here, the D/G-band ratios of GS and GS-Chi were 1.086 and 1.095, respectively. This difference might be due to the increased number of oxygenated groups at the upper and lower surfaces of GS after it was modified with Chi. In addition, the 2D band is usually used to confirm the presence of a few layers of GS. The value of the 2D band decreased as the number of layers GS increased. Here, the value of the 2D band of GS was slightly higher than the value of the 2D band of GS-Chi, which clearly indicates that Chi was successfully adsorbed onto the GS surface.

Figure 2C shows the structural features of GS-Chi. From the TEM image, GS-Chi, which was present as thin, wrinkled, rippled and flake-like structures, was readily observed. A TEM image of Chi-Au/Pt is shown in Fig. 2D. The obtained Chi-Au/Pt had a uniform globular morphology. Figure 2E shows the TEM image of GS-Chi-Au/Pt. Clearly, Au/Pt was bound to the GS. Here, Au^3+^ and Pt^2+^ ions were adsorbed by GS-Chi from aqueous solutions via chelation and then reduced to Au/Pt nanoparticles by Chi. GS-Chi-Au/Pt was formed, and Chi was used as a stabilizing agent and reducing agent. However, we found that the Au/Pt nanoparticles immobilized on the surface of GS-Chi became larger than the Au/Pt nanoparticles dispersed in the Chi solution. The potential explanation is that Chi agglomerated when a large amount Chi was adsorbed to the surface of GS, and the agglomeration of Chi would cause the Au/Pt nanoparticles to agglomerate. Figure 2F shows the HRTEM image of Chi-Au/Pt, and Fig. 2G–I shows the TEM elemental mapping image of Chi-Au/Pt. The diameter of Au/Pt was approximately 1–5 nm, and the Au and Pt elements were distributed uniformly randomly and relatively independently. Figure 2J shows the HRTEM image of GS-Chi-Au/Pt, and Fig. 2K–M shows the TEM elemental mapping image of GS-Chi-Au/Pt. The diameter of Au/Pt was approximately 60 nm, and Au and Pt elements were distributed uniformly randomly and centralized. In addition, the EDS spectrum of GS-Chi-Au/Pt confirmed the presence of Chi, Au, and Pt elements, indicating that Au/Pt had been loaded on the surface of GS. Cu was observed because the samples were fixed on the Cu network for testing.

### Electrochemical Characterization of the Immunosensor

Chronocoulometry was employed to investigate the effective surface areas of the bare GCE and GS-Chi-GCE (Additional file 1: Fig. S3). The effective surface areas of the electrode were calculated using Eq. (1), which was devised by Anson [36].

1 $$Q\left( t \right) = 2nFAcD^{1/2} t^{1/2} /\pi^{1/2} + Q_{{{\text{dl}}}} + Q_{{{\text{ads}}}}$$

Here, 0.1 mM K_3_[Fe(CN)_6_] was used as a model complex, and the diffusion coefficient *D* was 7.6 × 10^–6^ cm^2^ s^−1^. In Eq. (1), A is the effective surface area of the electrode, *Q*_dl_ is the double layer charge, *n*, *F* and *c* have their usual meanings, and *Q*_ads_ is the faradic charge. The effective surface areas of the bare GCE and GS-Chi-GCE were calculated to be 0.087 cm^2^ and 0.301 cm^2^ from the slope of the *Q* versus *t*^1/2^ curve (Additional file 1: Fig. S3B). The effective surface area of GS-Chi-GCE was 3.5 times larger than that of the bare GCE. The effective surface area of GCE increased substantially after modification with GS-Chi.

**Fig. 3:**
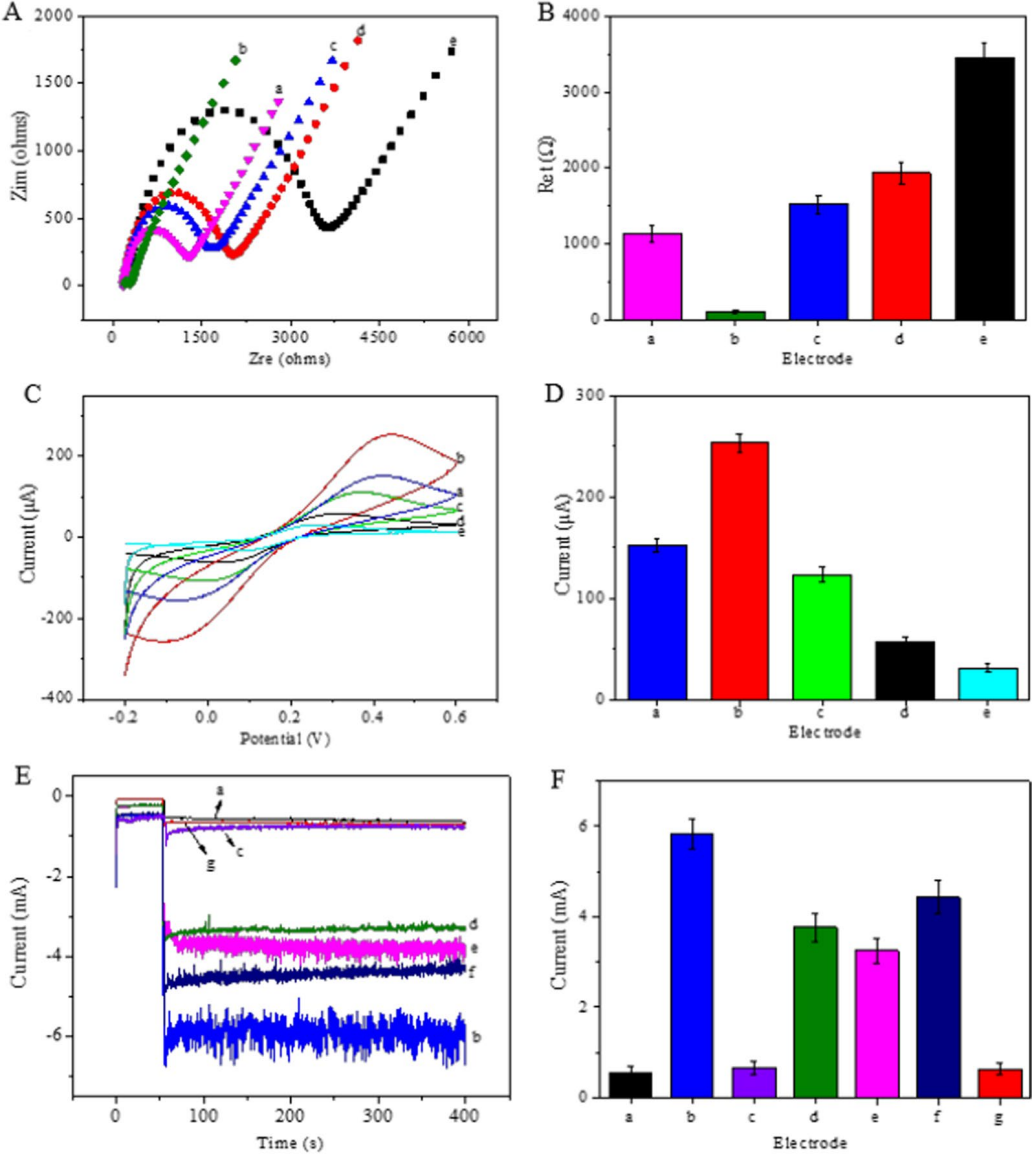
**A** Nyquist plots and **B** the *R*_et_ (error bar = RSD, *n* = 5) obtained from the EIS characterization of electrodes at different modification steps in electrolyte (pH 7.0) containing 5 mM [Fe(CN)_6_]^3−/4−^: (a) GCE, (b) GS-Chi-GCE, (c) AIV H9/MAbs-GS-Chi-GCE, (d) BSA-AIV H9/MAbs-GS-Chi-GCE, (e) AIV H9-BSA-AIV H9/MAbs-GS-Chi-GCE. **C** and **D** CV of (a) GCE, (b) GS-Chi-GCE, (c) AIV H9/MAbs-GS-Chi-GCE, (d) BSA-AIV H9/MAbs-GS-Chi-GCE and (e) AIV H9-BSA-AIV H9/MAbs-GS-Chi-GCE. The scan rate was 50 mV s^−1^. **E** and **F** Amperometric i–t curves and measured responses of the immunosensor for the detection of 10^6.37^ EID_50_/mL AIV H9 (a) without labels; (b) with GS-Chi-Au/Pt-AIV H9/PAbs, (c) GS-Chi-AIV H9/PAbs, (d) GS-Chi-Au-AIV H9/PAbs, (e) Chi-Au/Pt-AIV H9/PAbs and (f) GS-Chi-Au/Pt-AIV H9/PAbs as labels; and (g) without incubation with AIV H9 but with GS-Chi-Au/Pt-AIV H9/PAbs as labels at − 0.4 V towards the reduction of 10 mM H_2_O_2_ in electrolyte at pH 7.0

In this study, the step-by-step assembly process of the GCE was investigated by performing EIS in an electrolyte (pH 7.0) containing 5 mM [Fe(CN)6]^3−/4−^ and recorded from 0.01 Hz to 100 kHz using an amplitude of 10 mV at 0.2 V. Figure 3A shows Nyquist plots of EIS during electrode modification. In the Nyquist plots, the linear portion occurs at low frequencies and is related to electrochemical behaviour limited by diffusion. The semicircular portion occurs at high frequencies, and the semicircle diameter corresponds to the electron transfer resistance (*R*_et_) [37, 38]. The results are shown in Fig. 3A and B. The bare GCE exhibited a perfect semicircle (Fig. 3A, curve a) with a *R*_et_ of 1141 Ω (%RSD) = 2.97 (Fig. 3B, columnar a), while a very small semicircle (Fig. 3A, curve b) with a *R*_et_ of 103 Ω (%RSD) = 1.87 (Fig. 3B, columnar b) was observed after modification with GS-Chi because of the large electron transfer-promoting effect and excellent conductivity of GS. When AIV H9/MAbs, BSA and AIV H9 (10^6.37^ EID_50_/mL) were successively loaded onto the modified electrode, and the relevant *R*_et_ increased sequentially (1526 Ω (%RSD) = 2.73, 1934 Ω (%RSD) = 3.21 and 3434 Ω (%RSD) = 3.21, respectively) (Fig. 3B, columns c-e) because the resistance increased due to the poor conducting power of AIV H9/MAbs, BSA and AIV H9. These results revealed the successful construction of the modified electrode. The electron transfer rate constant (*K*_et_) was successfully obtained from Eq. (2), which shows the relationship between *R*_et_ and *K*_et_ [39], as follows:

2 $$K_{{{\text{et}}}} = \frac{{{\text{RT}}}}{{n^{2} F^{2} R_{{{\text{et}}}} AC_{{{\text{redox}}}} }}$$

A corresponds to the geometrical area of the electrode surface, while redox is the concentration of Fe(CN)_6_^4−/3−^. The *K*_et_ values were calculated to be 6.62 × 10^–5^ cm s^−1^, 73.69 × 10^–5^ cm s^−1^, 4.92 × 10^–5^ cm s^−1^, 3.87 × 10^–5^ cm s^−1^ and 2.18 × 10^–5^ cm s^−1^ for GCE, GS-Chi-GCE, AIV H9/MAbs-GS-Chi-GCE, BSA-AIV H9/MAbs-GS-Chi-GCE and AIV H9-BSA-AIV H9/MAbs-GS-Chi-GCE, respectively.

In addition, cyclic voltammetry (CV) is an effective technique for evaluating the successful modification of electrodes. The results are shown in Fig. 3C and D. The CV curve of the GCE electrode showed a reversible redox label of Fe(CN)_6_^4−/3−^, while the peak current of GS-Chi-GCE increased substantially due to the large electron transfer-promoting effect and excellent conductivity of GS. After AIV H9/MAbs, BSA and 10^6.37^ EID_50_/mL AIV H9 were immobilized on the modified electrode, the relevant peak current decreased in turn. The CV results were consistent with the EIS results, indicating the successful construction of the AIV H9 immunosensor.

Amperometric i–t curve measurements were employed to investigate the electrocatalytic performance of the proposed immunosensor. The results are shown in Fig. 3E and F. The electrocatalytic activity of GS-Chi (curve a) was ignored because it was weaker than that of GS-Chi-Au/Pt (curve b). During the immunosensor detection process, the electrocatalytic performance between GS-Chi-H9/PAbs, GS-Chi-Au-H9/PAbs, Chi-Au/Pt-H9/PAbs, GS-Chi-Pt-H9/PAbs and GS-Chi-Au/Pt-H9/PAbs as labels was compared. The immunosensors from the same batch were incubated with 10^6.37^ EID_50_/mL AIV H9 and then incubated with the two different types of labels. Finally, amperometric i–t measurements were performed in electrolyte (pH 7.0) with 10 mmol/L H_2_O_2_. As expected, the immunosensor using GS-Chi-Au/Pt-H9/PAbs (curve b) as the label displayed a much higher amperometric change than that using GS-Chi-H9/PAbs (curve c), GS-Chi-Au-H9/PAbs (curve d), Chi-Au/Pt-H9/PAbs (curve e) and GS-Chi-Pt-H9/PAbs (curve f) as the labels. The high sensitivity was mainly attributed to the large specific surface area, enhanced electrical conductivity and fast electron transport of GS and the excellent catalytic activity of Au/Pt. When no AIV H9 was immobilized on the electrode, the peak current was similar to curve a after the immunosensor was reacted with GS-Chi-Au/Pt-H9/PAbs (curve g), indicating that our developed immunosensor has good selectivity.

### Optimization of the Method

The following parameters were optimized: (a) GS-Chi concentration; (b) AIV H9/MAb concentration; (c) AIV H9/PAb concentration; (d) ratio of Au, Pt and GS; (e) pH value of the electrolyte; (f) H_2_O_2_ concentration; (g) incubation time of AIV H9; and (h) incubation time of GS-Chi-Au/Pt-AIV H9/PAbs bioconjugates. Respective descriptions and figures of optimization methods are provided in Electronic Supporting Material. Briefly, the following experimental conditions were observed to produce the best results: (a) the best concentration of GS-Chi was 1.0 mg/mL; (b) best concentration of AIV H9/MAbs was 10 µg/mL; (c) best concentration of AIV H9/PAbs was 0.5 µg/mL; (d) best ratio of Au, Pt and GS was 2:2:20 (Au (0.1 mg/mL), Pt (0.1 mg/mL) and GS (1 mg/mL)); (e) best pH value of the electrolyte was 7.0; (f) best concentration of H_2_O_2_ was 10 mM; (g) best incubation time of AIV H9 was 40 min; and (h) best incubation time of GS-Chi-Au/Pt-AIV H9/PAbs bioconjugates was 60 min.

### Detection of AIV H9 with the Immunosensor

**Fig. 4:**
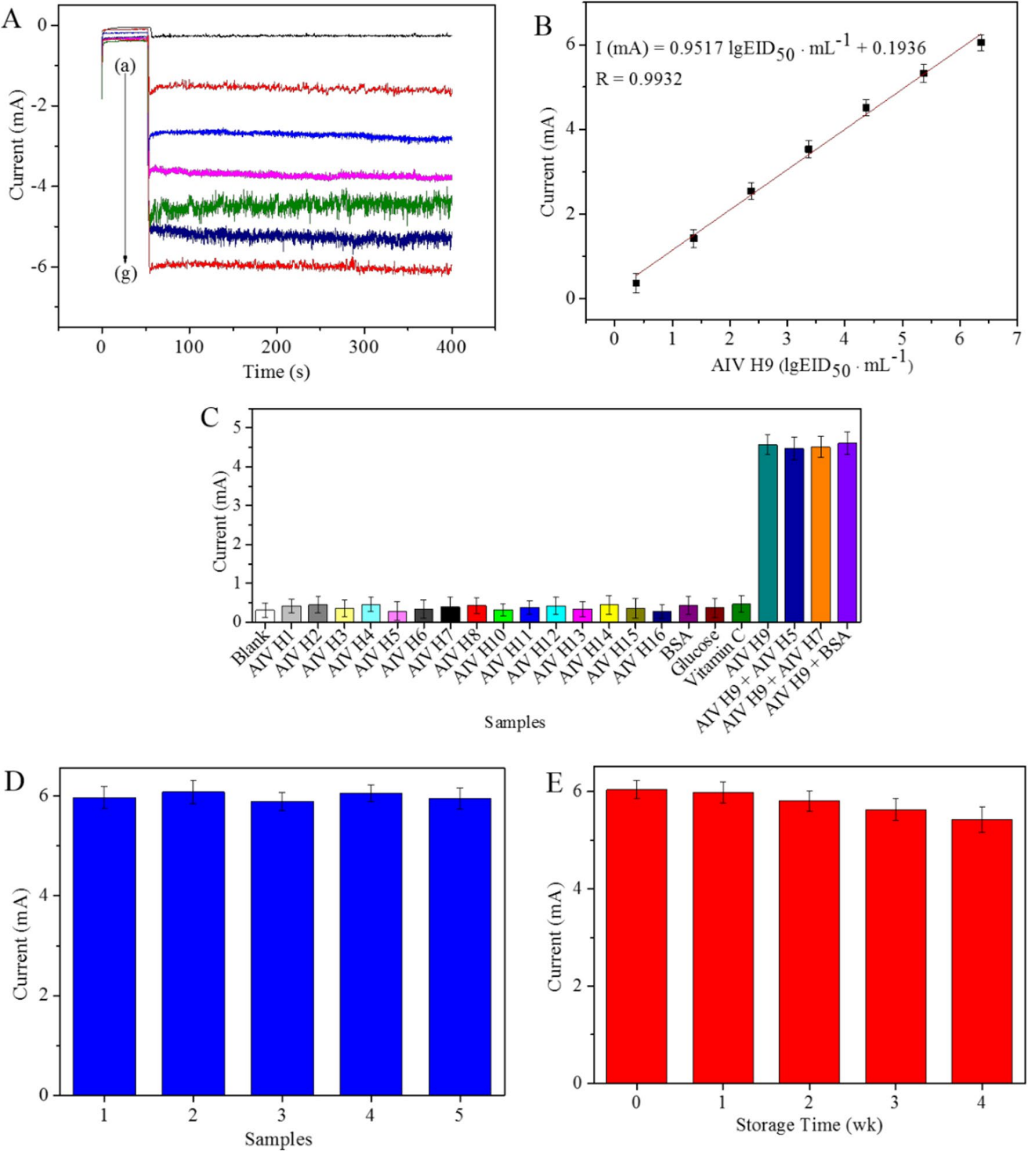
**A** Amperometric i–t current response of the immunosensor towards the addition of 10 mM H_2_O_2_ for detecting the following different concentrations of AIV H9 at − 0.4 V versus SCE: (a) 10^0.37^ EID_50_ mL^−1^, (b) 10^1.37^ EID_50_ mL^−1^, (c) 10^2.37^ EID_50_ mL^−1^, (d) 10^3.37^ EID_50_ mL^−1^, (e) 10^4.37^ EID_50_ mL^−1^, (f) 10^5.37^ EID_50_ mL^−1^ and (g) 10^6.37^ EID_50_ mL^−1^. **B** Calibration curve of the immunosensor response to different concentrations of AIV H9. Error bar = RSD (*n* = 5). **B** Calibration curve of the immunosensor response to different concentrations of AIV H9 (*n* = 5). **C** Specificity of the immunosensor towards the target AIV H9 and other interfering substances. **D** Reproducibility of the developed immunosensor in the presence of 10^6.37^ EID_50_ mL^−1^ AIV H9. **E** Long-term stability of the proposed immunosensor

Under the optimized reaction conditions, the detection capability of the proposed electrochemical immunosensor was determined by constructing an amperometric i–t curve at an initial potential of − 0.4 V in 10 mL of electrolyte (pH 7.0) containing 10 mM H_2_O_2_ to detect AIV H9 at a concentration ranging from 10^6.37^ EID_50_ mL^−1^ to 10^1.37^ EID_50_ mL^−1^. As shown in Fig. 4A, the current signal responses of the electrochemical immunosensor were enhanced with increasing concentrations of AIV H9. The difference in current signal intensity between the blank (curve a) and the AIV H9 curve indicated that the nonspecific adsorption on the electrochemical immunosensor and the effects of the background current of GS-Chi were negligible. The calibration plot showed a good linear relationship between the change in the current signal and the logarithm values of the AIV H9 concentration (Fig. 4B). The linear regression equation of the calibration curve was I (mA) = 0.9517 lgEID_50_ mL^−1^ + 0.1936, with a correlation coefficient of *R* = 0.9932 and a low detection limit of 10^0.82^ EID_50_ mL^−1^ (*S*/*N* = 3). In addition, the analytical performance of this immunosensor was superior to that of the previous methods developed for the detection of AIV H9 (Table 2). The reasons are attributable to the following aspects: (1) the GS-Chi-Au/Pt nanocomposites used as signal labels had superior electrocatalytic capability towards the reduction of H_2_O_2_ because the planar electric transport properties were enhanced by electron–electron correlation assistance, and (2) GS-Chi used as a matrix not only had remarkable electroconductivity that promoted electron transfer towards the surface of the electrode but also provided a large specific surface area to firmly bind AIV H9/PAbs, which improved the antigen–antibody reaction to detect AIV H9.

**Table 2:** Comparison of the proposed immunosensor with other methods for AIV H9 detection

Method	Detection time	Detection limit	References
Virus isolation	4 days	1 EID_50_ mL^−1^	[6]
RT–PCR	6 h	100 EID_50_ mL^−1^	[7]
Real-time RT–PCR	4 h	1 EID_50_ mL^−1^	[8]
RT-LAMP	3 h	10 copies per reaction	[9]
ELISA	2 h	10^−2.3^ TCID_50_	[10]
Proposed immunosensor	1.5 h	10^0.82^ EID_50_ mL^−1^	This study

*TCID*_*50*_ 50% Tissue culture infective dose

### Selectivity, Reproducibility and Stability of the Immunosensor

The selectivity of the immunosensor played a crucial role in detecting target samples without separation. Several interfering substances, including AIV subtype H1 (10^5.61^ EID_50_ mL^−1^), H2 (10^6.29^ EID_50_ mL^−1^), H3 (10^7.43^ EID_50_ mL^−1^), H4 (10^5.45^ EID_50_ mL^−1^), H5 (128 HAUs), H6 (10^6.19^ EID_50_ mL^−1^), H7 (256 HAUs), H8 (10^6.23^ EID_50_ mL^−1^), H10 (10^5.73^ EID_50_ mL^−1^), H11 (10^6.47^ EID_50_ mL^−1^), H12 (10^7.12^ EID_50_ mL^−1^), H13 (10^5.37^ EID_50_ mL^−1^), H14 (10^6.72^ EID_50_ mL^−1^), H15 (10^5.81^ EID_50_ mL^−1^), H16 (10^5.63^ EID_50_ mL^−1^), BSA (1.0 μg/mL), glucose (1.0 μg/mL) and vitamin C (1.0 μg/mL), were used in this work to evaluate the selectivity of the developed immunosensor. The results are shown in Fig. 4C. The current responses of the mixtures of AIV H9 (10^4.37^ EID_50_ mL^−1^) with other possible interfering substances were similar to those of AIV H9 (10^4.37^ EID_50_ mL^−1^) alone, while nonspecific substances and blank solution showed similar current responses, indicating that the proposed electrochemical immunosensor had high selectivity for AIV H9 detection.

The reproducibility of the proposed electrochemical immunosensor was investigated by recording the current responses in the presence of the same concentrations of 10^6.37^ EID_50_ mL^−1^ AIV H9. The results are shown in Fig. 4D. The RSD of the intra-assay reproducibility of the immunosensor, which was 1.6%, is shown as an error bar and indicated that the developed immunosensor has good reproducibility.

The long-term stability of the fabricated electrochemical immunosensor was further investigated by storing BSA-AIV H9/MAbs-GS-Chi-GCE at 4 °C when not in use and then successively incubating it with AIV H9 (10^6.37^ EID_50_ mL^−1^) and GS-Chi-Au/Pt-AIV H9/PAbs bioconjugates. As shown in Fig. 4E, the current responses decreased by less than 4% after 2 weeks of storage. The current responses eventually retained 89.72% of the initial value. Based on the results, the long-term stability of the proposed electrochemical immunosensor for AIV H9 detection was acceptable.

### Analysis of AIV H9 in Clinical Samples

Ninety-eight clinical samples were obtained from chickens with the permission of the host of the live poultry markets, and clinical samples were detected using the developed immunosensor. Three AIV H9-positive samples were identified. The results were confirmed by virus isolation. The results are shown in Table 3a and b. All the results obtained from the clinical samples detected using the proposed immunosensor were consistent with the results obtained through virus isolation [40].

**Table 3:** Results from clinical samples (a); results from the analysis of positive samples (b); and results for the recovery of different concentrations of AIV H9 from clinical samples (c)

(a) Method	Total number of samples	Number of positive samples	Positivity rate (%)
Proposed immunosensor	98	3	3.06
Virus isolation	98	3	3.06

(b) No	Results from the proposed immunosensor			Results of virus isolation
	Measured concentration (EID_50_ mL^−1^)	Average (EID_50_ mL^−1^)	RSD (%) (*n* = 5)	
1	60.24, 57.31, 62.17, 63.54, 64.49	61.55	4.63	Positive
2	141.25, 137.86, 143.74, 136.47, 142.18	140.30	2.17	Positive
3	192.67, 188.34,193.28, 187.59, 195.73	191.52	1.80	Positive

(c) No	Initial AIV H9 concentration in the sample (EID_50_ mL^−1^)	Added amount of AIV H9 (EID_50_ mL^−1^)	Total found		Recovery rate (%) (*n* = 5)
			Average (EID_50_ mL^−1^)	RSD (%) (*n* = 5)	
1	61.55	100.00	169.03	3.47	104.63
2	61.55	150.00	206.93	2.73	97.82
3	140.30	200.00	326.74	4.07	96.02
4	140.30	250.00	381.37	2.17	97.71
5	191.52	300.00	497.31	4.18	101.18
6	191.52	400.00	584.62	3.75	98.83

Standard spiking was also employed to evaluate the accuracy and practical applicability of the developed immunosensor. A series of different concentrations of AIV H9 (100.00, 150.00, 200.00, 250.00, 300.00 and 400.00 EID_50_ mL^−1^) was added to the three AIV H9-positive samples (from the aforementioned clinical samples that were identified as the AIV H9-positive sample), followed by detection using the fabricated electrochemical immunosensor according to the steps listed above in “Electrochemical Measurement” section. The recovery was calculated as the ratio between the Found and Added AIV H9. The results are shown in Table 3c. The recoveries were obtained from 96.02 to 104.63% with an RSD of 2.17–4.18% (*n* = 5), indicating that the developed immunosensor was feasible for AIV H9 detection in clinical samples.

## Conclusions

In this study, we synthesized Au/Pt nanoparticle-functionalized GS (GS-Chi-Au/Pt) with high electrocatalytic activity towards H_2_O_2_ reduction. We have developed an enzyme-free sandwich electrochemical immunosensor utilizing GS-Chi as the matrix platform and GS-Chi-Au/Pt as the signal amplification label. This assay system is capable of detecting AIV H9 in clinical samples. Benefiting from the favourable cooperation of GS-Chi-Au/Pt with good H_2_O_2_ catalysis, ideal conductivity of GS-Chi and efficient antibody immobilization, the established immunosensor exhibited good sensitivity, specificity, reproducibility, accuracy and stability. Importantly, this developed method not only expands the application of GS-Chi-Au/Pt in electrochemical biosensors but also provides a novel nonenzymatic method for the accurate determination of other biomolecules in clinical diagnosis.

## Supplementary Information

**Additional file 1.** Appendix A. Supplementary data.

## Data Availability

All data generated or analysed during this study are included in this article and its supplementary information file.

## Abbreviations

Chi: Chitosan
GS: Graphene sheets
GS-Chi-Au/Pt: Chitosan-modified graphene sheet-functionalized Au/Pt nanoparticles
AIVH9: Avian influenza virus H9 subtype
GS-Chi: Graphene–chitosan
AIVH9/MAb: Avian influenza virus H9-monoclonal antibody
AIVH9/MAb: Avian influenza virus H9-monoclonal antibody
H_2_O_2_: Hydrogen peroxide
BSA: Bovine serum albumin
ddH_2_O: Double-distilled deionized water
PBS: Phosphate-buffered saline
GVRI: Guangxi Veterinary Research Institute
UHK: University of Hong Kong, China
PU: Pennsylvania State University, USA
UCONN: University of Connecticut, USA
CIVDC: China Institute of Veterinary Drug Control
HAUs: Haemagglutination units
FT-IR: Fourier-transform infrared
GCE: Glassy carbon electrode
SCE: Saturated calomel electrode
TEM: Transmission electron microscopy
EDS: Energy-dispersive X-ray spectroscopy
EIS: Electrochemical impedance spectroscopy
R_et_: Electron transfer resistance
K_et_: Electron transfer rate constant
CV: Cyclic voltammogram
TCID_50_: 50% tissue culture infective dose
