# Supplementary material for: An Enzyme-Free Sandwich Amperometry-Type Immunosensor Based on Au/Pt Nanoparticle-Functionalized Graphene for the Rapid Detection of Avian Influenza Virus H9 Subtype
Source: Nanoscale Res Lett. 2022 Nov 21;17:110. doi: 10.1186/s11671-022-03747-8 (PMC9676155; doi:10.1186/s11671-022-03747-8)
Supplement: Supplementary file 1 — Additional file 1. Appendix A. Supplementary data. [file 11671_2022_3747_MOESM1_ESM.docx]

**Additional file 1**

**An enzyme-free sandwich amperometry-type immunosensor based on Au/Pt nanoparticle-functionalized graphene for the rapid detection of avian influenza virus H9 subtype**

Jiaoling Huang, Zhixun Xie^*^, Meng Li, Sisi Luo, Xianwen Deng, Liji Xie, Qing Fan, Tingting Zeng, Yanfang Zhang, Minxiu Zhang, Sheng Wang, Zhiqin Xie, Dan Li

Guangxi Key Laboratory of Veterinary Biotechnology, Key Laboratory of China (Guangxi)-ASEAN Cross-border Animal Disease Prevention and Control, Ministry of Agriculture and Rural Affairs of China, Guangxi Veterinary Research Institute, Nanning, Guangxi, China

***Correspondence**

Zhixun Xie, 51 You Ai North Road, Nanning, 530001 Guangxi, China. E-mail: [xiezhixun@126.com](mailto:xiezhixun@126.com)

***Cyclic voltammetry of GS-Chi-Au/Pt***

Cyclic voltammetry was employed to investigate the electrocatalytic mechanism of the proposed sandwich-type immunosensors. Fig. S1 presents the cyclic voltammogram of the proposed immunosensor for the detection of 10^6.37^ EID_50_/mL AIV H9 using GS-Chi-Au/Pt-AIV H9/PAbs as labels in electrolyte at pH=7.0 before (curve a) and after (curve b) the addition of 10 mM H_2_O_2_. Before the addition of H_2_O_2_, cyclic voltammetry of the proposed immunosensor did not show any obvious reduction peak (curve a), and a dramatic increase in the reduction current was observed at -0.4 V after addition of H_2_O_2_ (curve b). The results showed the good electrocatalytic performance of the proposed immunosensor towards the reduction of H_2_O_2_, demonstrating that amperometric i-t measurements were performed in electrolyte at pH=7.0 with a working potential of -0.4 V would be beneficial to minimize the responses of common interfering species and decrease the background current.

Fig. S1. Cyclic voltammetry (CV) of the GS-Chi-Au/Pt-modified electrode in electrolyte at pH=7.0 before (a) and after (b) the addition of 10 mM H_2_O_2_.

***Optimization of method***

The concentration of GS-Chi is a critical factor because it plays an important role in immobilization of AIV H9/MAbs and enhancing electron transfer; in other words, the concentration of GS-Chi will affect the amperometric response. Therefore, optimization of the concentration of GS-Chi is necessary. Fig. S2A shows currents of the amperometric i-t curve when the GCE was modified with different concentrations of GS-Chi to detect 10^6.37^ EID_50_/mL AIV H9 using GS-Chi-Au/Pt-AIV H9/PAbs as labels in electrolyte (pH=7.0) containing 10 mM H_2_O_2_. As shown in this figure, a gradual increase in GS-Chi concentrations from 0.25 to 1.0 mg/mL resulted in remarkable increases in the current response. During this period, the GS-Chi increased, resulting in an abundance of AIV H9/MAbs anchoring sites on the surface of the electrode and a gradual increase in current responses. Nevertheless, the current response signal decreased as the GS-Chi concentration was increased from 1 to 2 mg/mL. These results indicated that 1.0 mg/mL GS-Chi was an optimal condition. The current changes indicated that a suitable concentration of GS-Chi can efficiently enhance the conductivity, while excessive GS-Chi was loaded on the GCE as a substrate, which increased the interface electron transfer resistance of the electrode.

Concentrations ranging from 1 to 20 µg/mL and 0.1 to 1.5 µg/mL were investigated for AIV H9/MAbs and AIV H9/PAbs, respectively. The current response gradually increased with increasing AIV H9/MAbs (Fig. S2B) and AIV H9/PAbs concentrations (Fig. S2C) and then reached maximum values at 10 µg/mL and 0.5 µg/mL, respectively. The plateau signal occurred because of oversaturation of the electrode surface, resulting in a limited number of conjugated AIV H9/MAbs/AIV H9/AIV H9/PAbs complexes that could be formed on the surface of the electrode. Thus, 10 and 0.5 µg/mL were the optimal concentrations for AIV H9/MAbs and AIV H9/PAbs, respectively.

Fig. S2D and E display the effect of the ratio of Au, Pt and GS (Au:Pt:GS) in GS-Chi-Au/Pt-AIV H9/PAbs on the current responses of the electrochemical immunosensor. The results showed that the signal responses increased with the ratio of Au, Pt and GS ranging from 1:0:20 to 4:0:20 in the absence of Pt and 0:1:20 to 0:4:20 in the absence of Au. In the presence of Au and Pt, the signal responses increased with the ratio of Au, Pt and GS in the range from 1:1:20 to 2:2:20, and then the signal responses levelled off. In addition, Au and Pt together exhibit much higher catalytic ability than their monometallics because of the synergistic effect. Therefore, the ratio (2:2:20) of Au (0.1 mg/mL), Pt (0.1 mg/mL) and GS (1 mg/mL) was chosen as the optimized signal label composition in this study.

The pH value of the electrolyte is an important factor for electrochemical immunosensors. Fig. S2F shows the effect of the pH value in the electrolyte on the current responses. The current responses were enhanced with increasing pH values from 6.0 to 7.0 and then decreased when the pH value was above 7.0. The optimal amperometric response was achieved at pH=7.0. The reason for the fluctuation of the signal response may be that alkaline or acidic environments disrupt the stability of antibody-antigen binding. Thus, pH = 7.0 in the electrolyte was selected for use in subsequent experiments.

Fig. S2G shows the result of optimization of the concentration of H_2_O_2_. As shown in this figure, the current responses were enhanced when the H_2_O_2_ concentration was increased from 1 to 10 mM, and then the current responses levelled off after 10 mM. This is because 10 mM H_2_O_2_ reaches saturation, and excessive H_2_O_2_ will not join in the catalytic reaction. Therefore, the optimal concentration of H_2_O_2_ was 10 mM. In addition, the incubation time is a great influencing factor for both the antigen and the antibody. As shown in Fig. S2H and I, the current responses gradually increased with the incubation time of AIV H9 and GS-Chi-Au/Pt-AIV H9/PAbs until the immunoreaction finished at 40 min and 60 min, respectively. The results indicated that the immunoreaction of AIV H9/MAbs with AIV H9 will be finished after 40 min, and the immunoreaction of AIV H9 with GS-Chi-Au/Pt-AIV H9/PAbs will be finished after 40 min. Therefore, 40 min and 60 min were chosen as the optimal incubation time points for AIV H9 and GS-Chi-Au/Pt-AIV H9/PAbs, respectively.


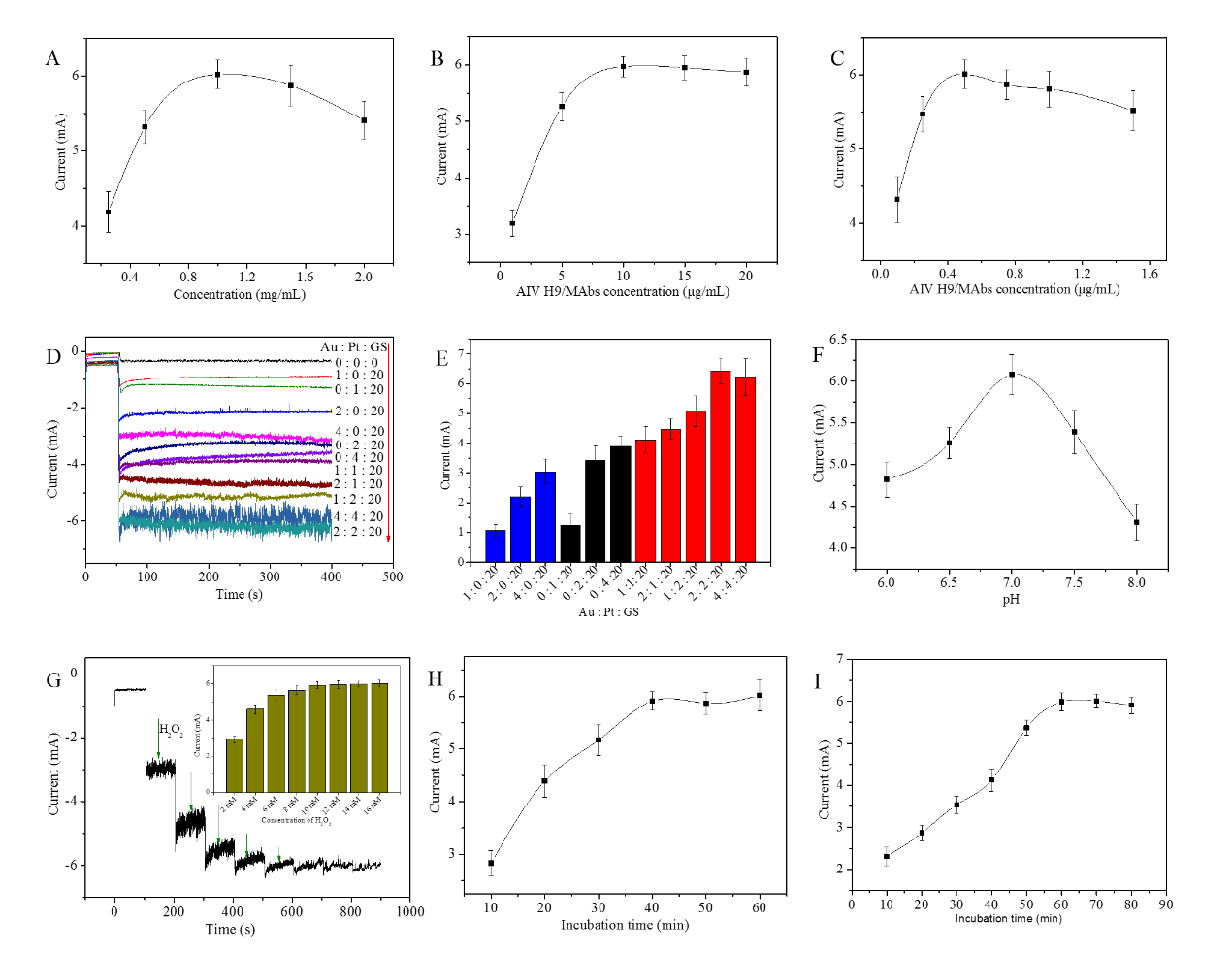


Fig. S2. Optimizations of (A) GS-Chi concentration (from 0.25 to 2 mg/mL) and (B) AIV H9/MAbs concentration (from 1 to 20 µg/mL). (C) AIV H9/PAbs concentration (from 0.1 to 1.5 µg/mL), (D, E) ratio of Au, Pt and GS, (F) pH values of electrolyte (working buffer), (G) the concentration of H_2_O_2_, (H) incubation time of AIV H9, (I) incubation time of GS-Chi-Au/Pt-AIV H9/PAbs bioconjugates. The target AIV H9 was 10^6.37^ EID_50_·mL^-1^ in the optimization experiments. Error bar = RSD (n=5).


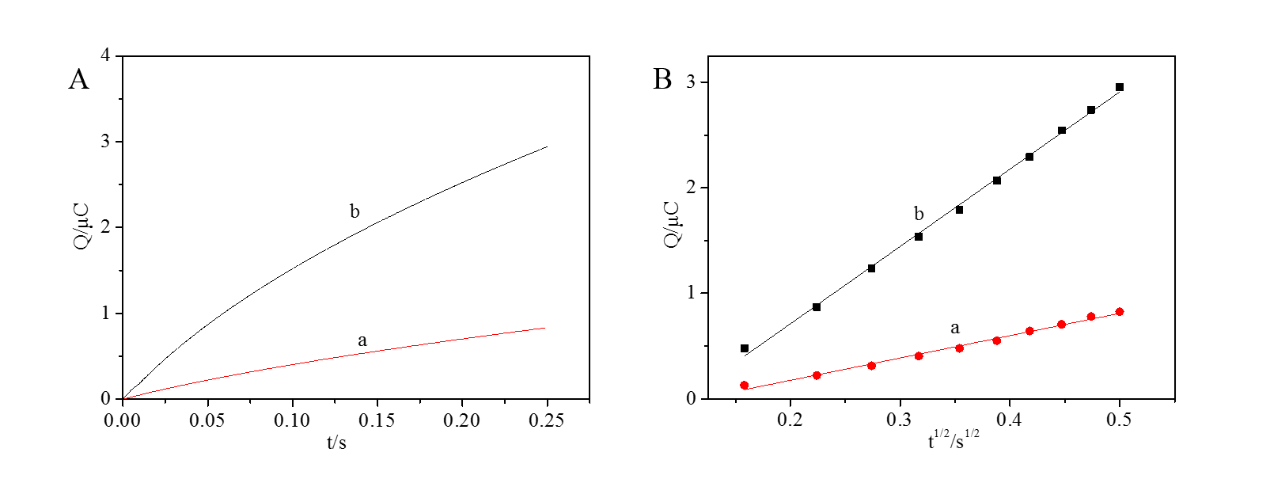


Fig. S3. (A) Plot of Q–t curve of the GCE (a) and GS-Chi-GCE (b) in 0.1 mM K_3_[Fe(CN)_6_] containing 1.0 M KCl. (B) Corresponding plot of Q–t^1/2^ on GCE (a) and GS-Chi-GCE (b).
